# Supplementary material for: Lipidomics analysis of juveniles’ blue mussels (Mytilus edulis L. 1758), a key economic and ecological species
Source: PLoS One. 2020 Feb 21;15(2):e0223031. doi: 10.1371/journal.pone.0223031 (PMC7034892; doi:10.1371/journal.pone.0223031)
Supplement: S1 Protocol — (DOCX) [file pone.0223031.s001.docx]

**S1_Protocol: Details of sample preparation procedures employed during spat and diet lipid extraction.**

Aliquots (10 mg dry weight - DW) of dry microalgae powder were homogenised via probe sonication in ice for 1 minute at max power with the addiction of 200 µl of milliQ water. For spat (10 mg DW), homogenization was completed by pestling in a glass mortar for 1 minute in ice with the addiction of 200 µl of milliQ water.

The homogenates were extracted according to Folch *et al.* (1957). Briefly 6 ml of 2:1 Chloroform: Methanol (FisherBrand) mixture were added to the homogenate, which was vigorously shaken and left to extract for 1 hour on ice. To avoid oxidation during the extraction, the vials were flushed with a gentle stream of nitrogen and kept away from direct light during all the extraction procedure. The mixture was centrifuged (2500 rpm 5 min. 4°C) and the supernatant recovered on a clean tube. The extraction was repeated on the pellet with a further 3 ml of Folch extraction mixture. Following a second centrifugation step the two organic supernatants were mixed together and a 0.9% KCl solution (VWR) was added to obtain a final ratio of 2:1:0.8 chloroform:methanol:0.9% KCl. The aqueous and organic phases were separated by a second centrifugation step (2500 rpm 5 mins 4°C). The organic layer was recovered and evaporated under a gentle nitrogen stream (NVap, Organomation). The dried lipid extracts were weighted to the 0.00001 g (Sartorius) and resuspended in 0.5 ml of chloroform constituting the total lipid extract (TLE). The TLE was divided into 2 sub-aliquots. One aliquot (400µl) was dried down in nitrogen and stored at -80 °C for lipid class and lipidomics analysis. To the second aliquot (100 µl) was added an internal standard (FA 17:0, Sigma + 0.001% of BHT, Cayman Chemical Company at the 10% of the total lipid mass) and processed for fatty acid methyl esters (FAME) analysis.
