# Supplementary figures and images for: Lipidomics analysis of juveniles’ blue mussels (Mytilus edulis L. 1758), a key economic and ecological species
Source: PLoS One. 2020 Feb 21;15(2):e0223031. doi: 10.1371/journal.pone.0223031 (PMC7034892; doi:10.1371/journal.pone.0223031)

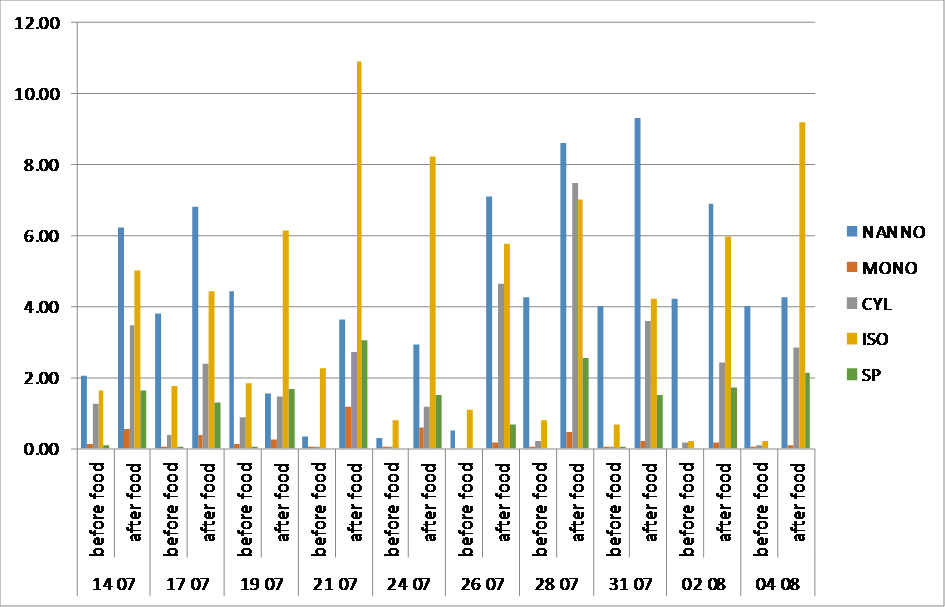

Supplement: S1 Fig — (TIF) [file pone.0223031.s002.tif]

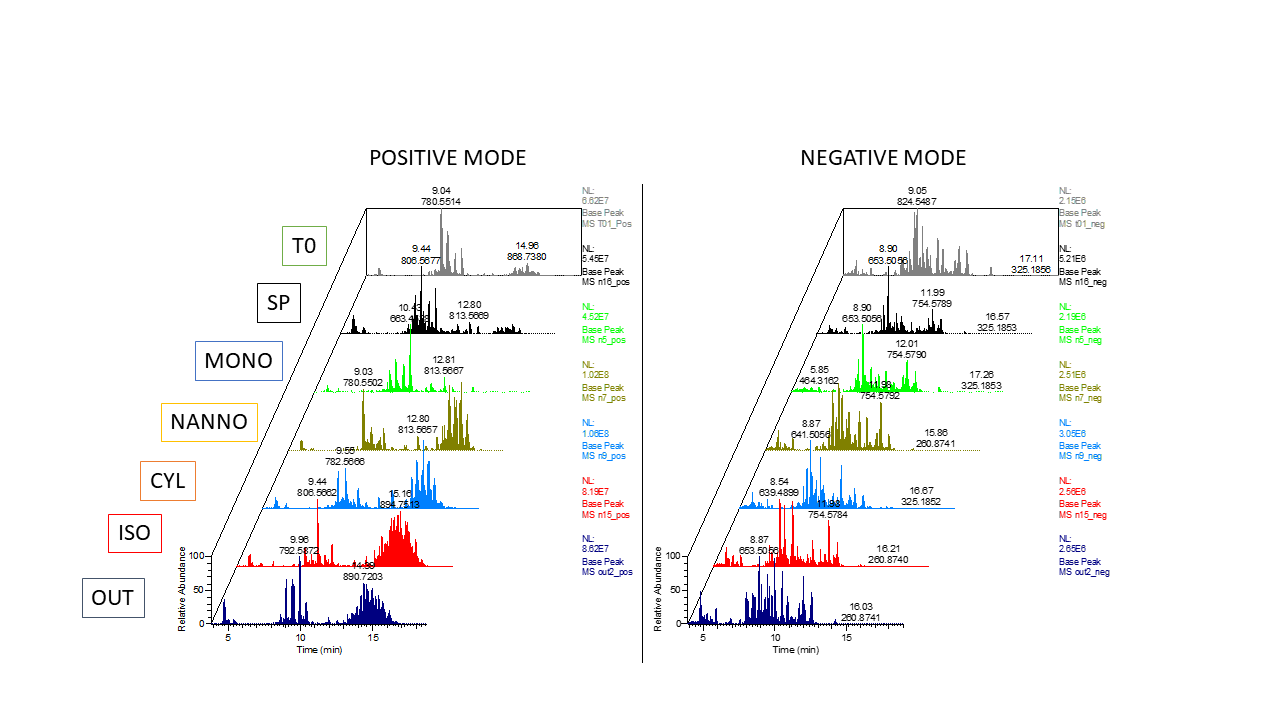

Supplement: S2 Fig — ESI POS mode profiles left traces, NEG mode profiles right traces. Data are aquired at precursor ion MS (MS’) via high resolution LC-MS platform (Exactive, ThermoScientific). Plotted via Excalibur 4.1 (ThermoScientific). (TIF) [file pone.0223031.s003.tif]

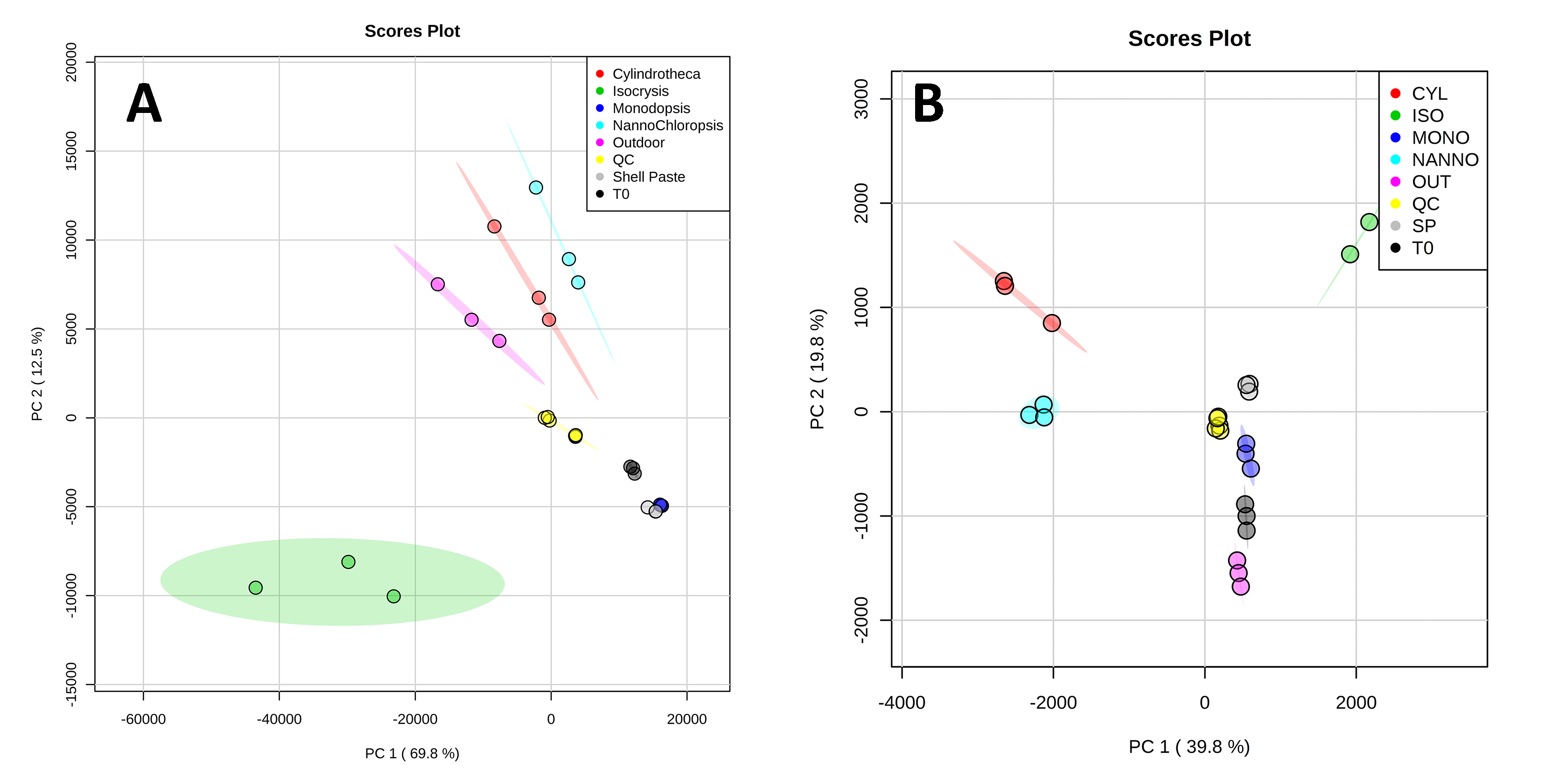

Supplement: S3 Fig — Principal Component Analysis of POS (A) and NEG (B) lipidomics spat dataset Plotted via MetaboAnalystR. CYS Cylindrotheca fusiformis fed spat, ISO Isocrysis galbana fed spat, MONO Monodopsis subterranean fed spat, NANNO Nannochloropsis oceanica fed spat, OUT Outdoor deployed spat, QC: Quality control samples, SP ShellPaste fed spat, T0: T0 samples. (TIF) [file pone.0223031.s004.tif]

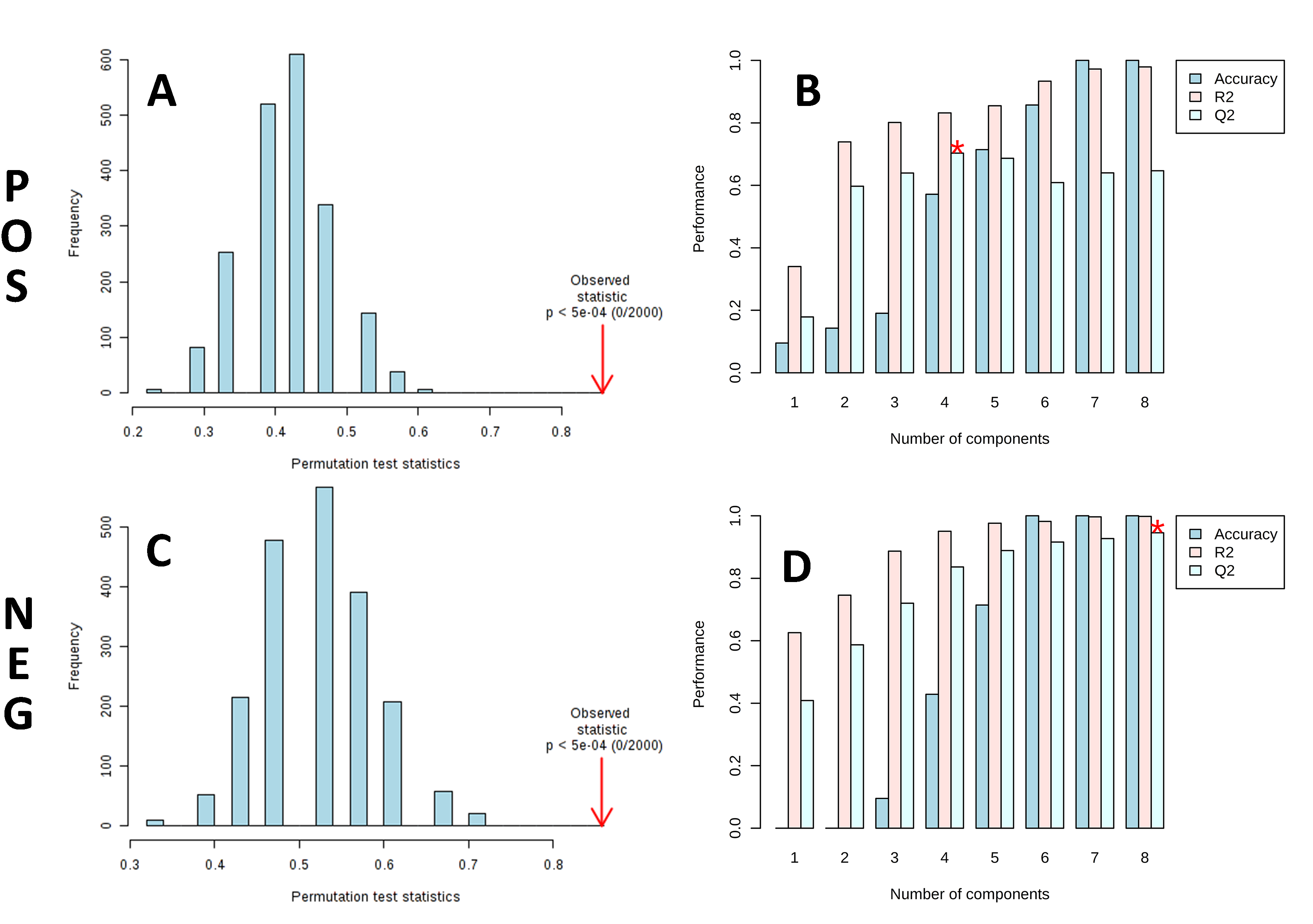

Supplement: S4 Fig — A: 2000-fold Permutation test POS data. B: 10-fold leave one out–Cross-validation analysis (LOOCV) for POS Data. Q2 used as parameter of model fitting. C: 2000-fold Permutation test NEG data. B: 10 LOOCV for NEG Data. Q2 used as parameter of model fitting. Plotted and calculated via MetaboAnalystR. (TIF) [file pone.0223031.s005.tif]

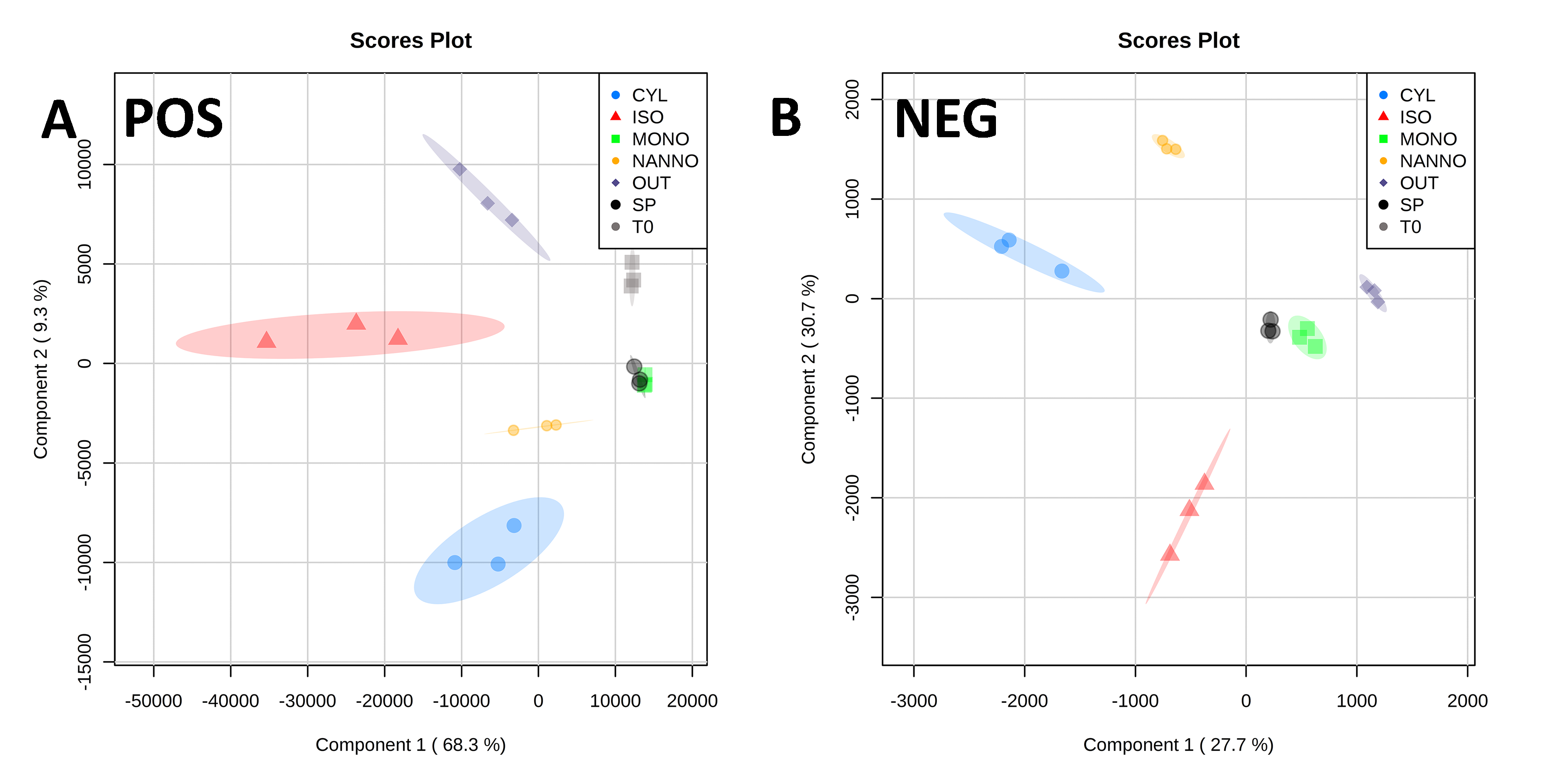

Supplement: S5 Fig — Partial least squares discriminant analysis (PLS-DA) plots of untargeted lipidomics data acquired in POS (A) and NEG (B). Full data is used in this model including unknown features. Plotted via MetaboAnalystR. (TIF) [file pone.0223031.s006.tif]

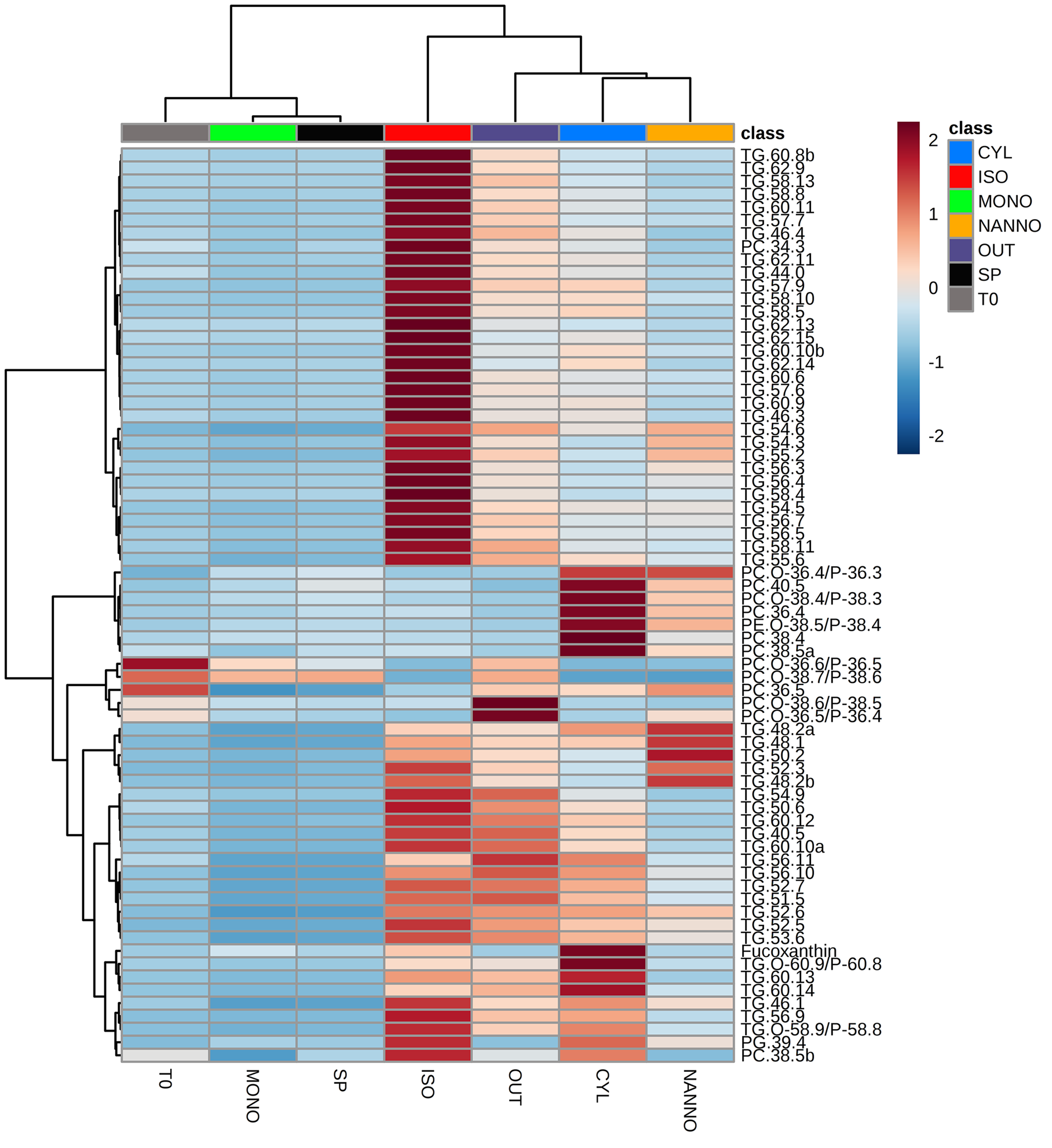

Supplement: S6 Fig — Full data is here used, including unknown features. Euclidean distance was distance measure, Ward as clustering algorithm. Lipids are rported for average in each group. Lipids are reported as class, n° carbon and n° of double bonds (e.g. TG.58.10). Colour coding for lipid expression from Blue (Low) to red (High). (TIF) [file pone.0223031.s007.tif]

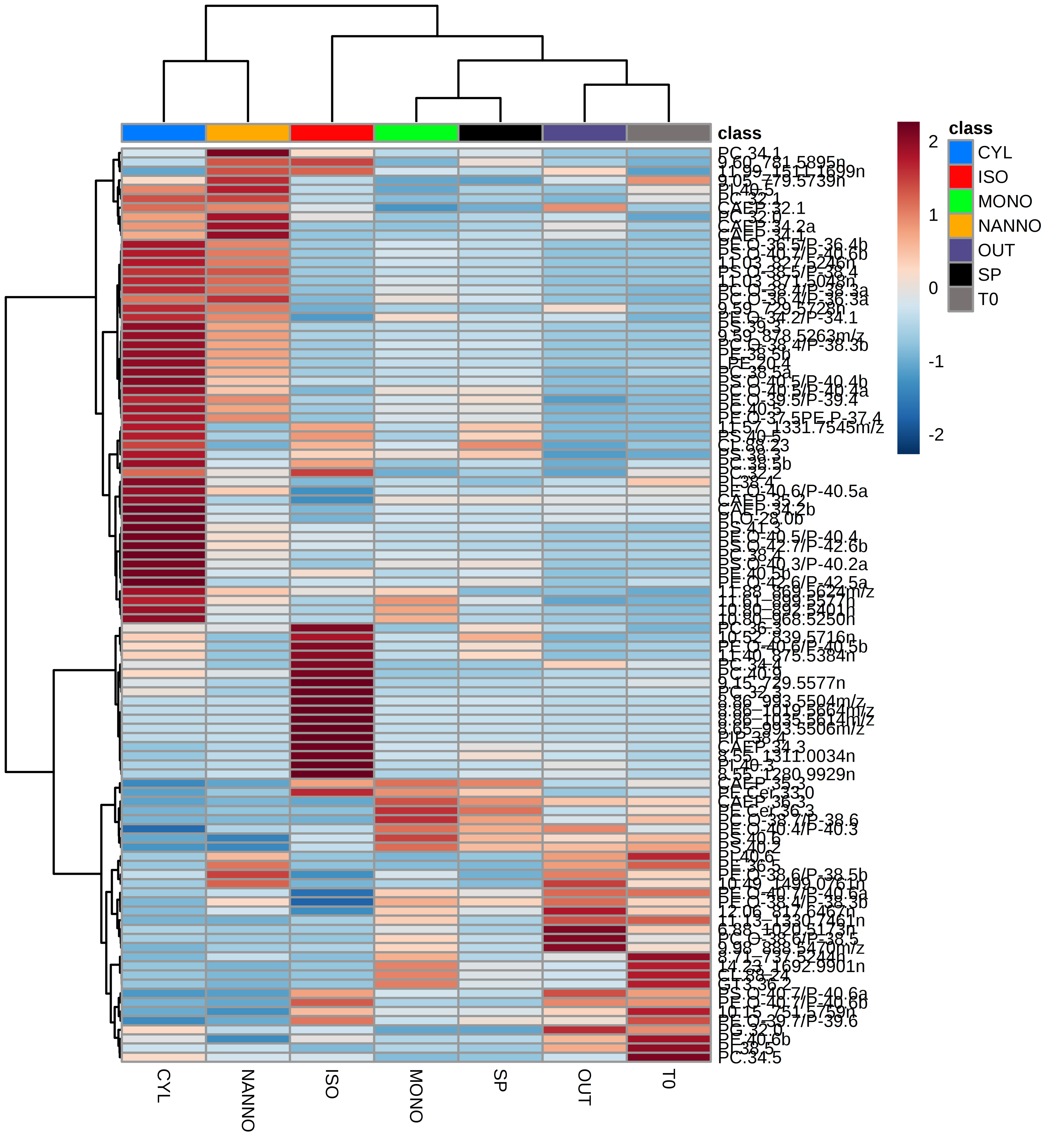

Supplement: S7 Fig — Full data is here used, including unknown features. Euclidean distance was distance measure, Ward as clustering algorithm. Lipids are rported for average in each group. Lipids are reported as class, n° carbon and n° of double bonds (e.g. TG.58.10). Colour coding for lipid expression from Blue (Low) to red (High). In absence of an exact mass ID features are reported as ret. time_mass/charge (e.g. 8.86_1019.5664m/z) or ret. time_neutral mass (e.g. 11.13_1330.7461n). (TIF) [file pone.0223031.s008.tif]

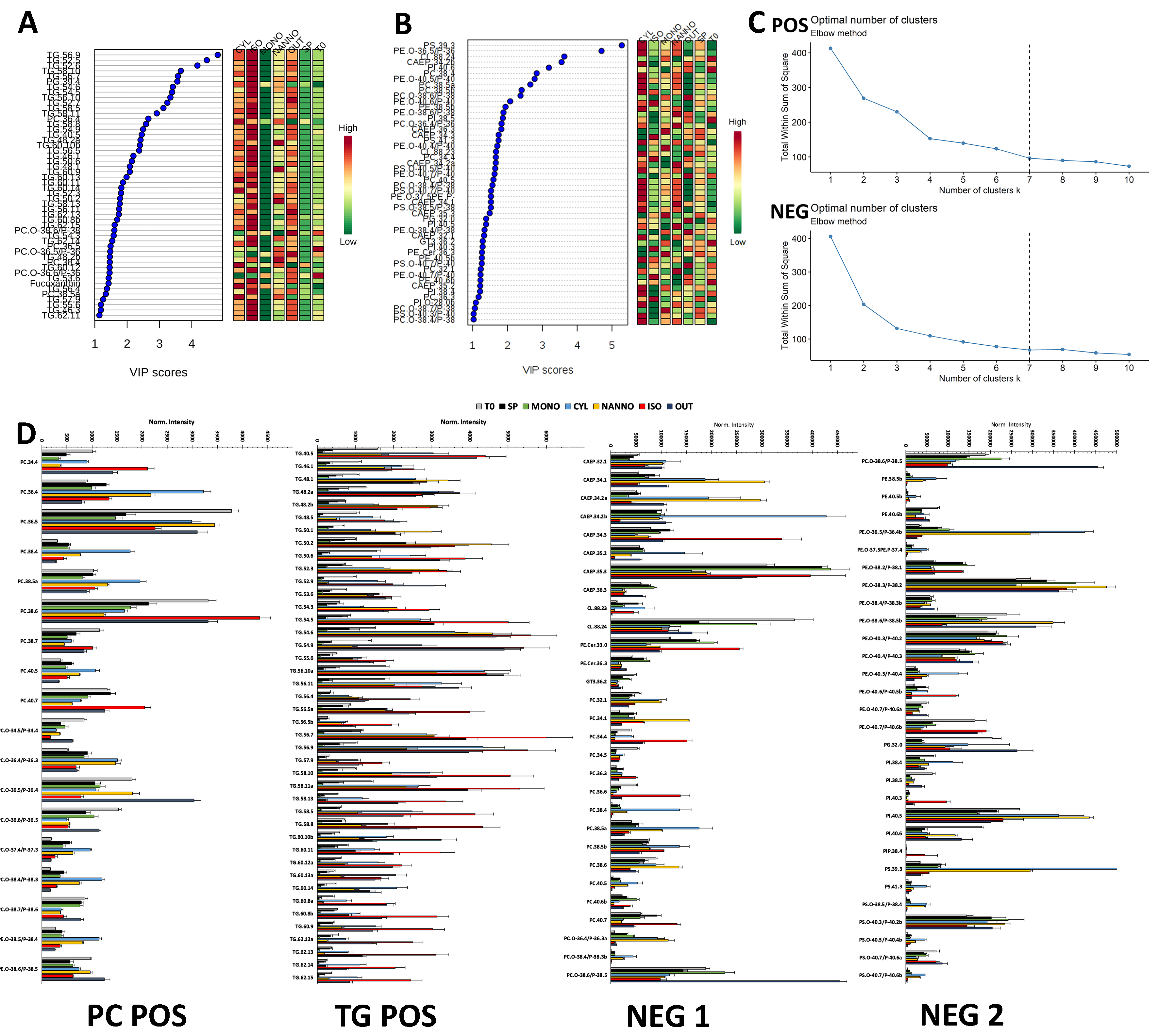

Supplement: S8 Fig — A: VIP score plot resulting from PLS-DA analysis of POS dataset. B: VIP score plot resulting from PLS-DA analysis of NEG dataset. C: Evaluation of optimal number of clusters via the “elbow method”. Top POS data, Bottom: NEG data. D: Histogram plots showing the the raw intensity of VIP evidenced by PLS-DA in POS (PC POS and TG POS) and NEG data (NEG1 and NEG2). NEG1 included lipids belonging to CAEP, CL, PE-Cer and PC; NEG 2 reports lipids belonging to PE, PI and PS. VIP score plots were calculated and plotted via ‘MetaboanlystR’ package [1], whereas the number of meaningful HC were calculated and plotted via the R package ‘factoextra’ [2]. Data in D are reported as average of Normalised intensity ± SD and Plotted via Daniel’s XL toolbox for Microsoft Excel. References S8 Fig. 1. Xia J, Chong J. MetaboanlystR: An R package for comprehensive analysis of metabolomics data. 0.0.0.9000 ed2018. 2. Kassambara A, Mudt F. Package 'factoextra'. 1.0.5 ed2017. (TIF) [file pone.0223031.s009.tif]
