# Supplementary material for: Lipidomics analysis of juveniles’ blue mussels (Mytilus edulis L. 1758), a key economic and ecological species
Source: PLoS One. 2020 Feb 21;15(2):e0223031. doi: 10.1371/journal.pone.0223031 (PMC7034892; doi:10.1371/journal.pone.0223031)
Supplement: S1 Table — (DOCX) [file pone.0223031.s010.docx]

**S1 Table: Main adducts and exact masses (for positive and negative ionization) of the lipid standard mixture used for exact mass (MS’) identification of lipidomics data.**

| **Lipid Class** | **Positive ionization** | | **Negative ionization** | |
| --- | --- | --- | --- | --- |
|  | **m/z** | **Main adduct** | **m/z** | **Main adduct** |
| ***FA (16:0)*** | n/a | n/a | 255.233 | **[M-H]^-^** |
| ***LPC (16:0)*** | 496.3398 | **[M+H]^+^** | 540.3307 | **[M+FA-H]^-^** |
| ***PS(14:0)_2_*** | 680.4497 | **[M+H]^+^** | 678.4352 | **[M-H]^-^** |
| ***PC(14:0)_2_*** | 678.5068 | **[M+H]^+^** | 722.4978 | **[M+FA-H]^-^** |
| ***PE(16:0)_2_*** | 692.5225 | **[M+H]^+^** | 690.5079 | **[M-H]^-^** |
| ***PG(14:0)_2_*** | 684.481 | **[M+NH_4_]^+^** | 665.4399 | **[M-H]^-^** |
| ***PA(16:0/18:1)*** | n/a | n/a | 673.4814 | **[M-H]^-^** |
| ***PI (16:0/16:0)*** | 828.5596 | **[M+NH_4_]^+^** | 809.5185 | **[M-H]^-^** |
| ***SM(d18:1/17:0)*** | 717.5905 | **[M+H]^+^** | 761.5814 | **[M+FA-H]^-^** |
| ***Cer(d18:1/16:0)*** | 520.5094 | **[M-H20+H]** | 536.5048 | **[M-H]^-^** |
| ***GluCer(d18:1/24:1)*** | 832.6637 | **[M+Na]^+^** | 854.6727 | **[M+FA-H]^-^** |
| ***MAG(16:0)*** | 353.2662 | **[M+Na]^+^** | n/a | n/a |
| ***PE-Cer(d16:1/24:0)*** | 745.6218 | **[M+H]^+^** | 743.6072 | **[M-H]^-^** |
| ***MAG(18:0)*** | 381.2975 | **[M+Na]^+^** | n/a | n/a |
| ***DAG(16:0)_2_*** | 591.4959 | **[M+Na]^+^** | n/a | n/a |
| ***DAG(18:0)_2_*** | 647.5585 | **[M+Na]^+^** | n/a | n/a |
| ***TAG(18:1)_3_*** | 902.8171 | **[M+NH_4_]^+^** | n/a | n/a |
| ***ChoE (16:0)*** | 647.5737 | **[M+Na]^+^** | n/a | n/a |
| ***CL(18:1)_4_*** | 1475.0687 | **[M+NH_4_]^+^** | 1456.0276 | **[M-H]^-^** |
| ***GM1(d18:1/18:0)*** | 1546.8839 | **[M+H]^+^** | 1544.8698 | **[M-H]^-^** |
| ***GM1(d18:1/20:0)*** | 1574.9152 | **[M+H]^+^** | 1572.9017 | **[M-H]^-^** |
