# Supplementary material for: Lipidomics analysis of juveniles’ blue mussels (Mytilus edulis L. 1758), a key economic and ecological species
Source: PLoS One. 2020 Feb 21;15(2):e0223031. doi: 10.1371/journal.pone.0223031 (PMC7034892; doi:10.1371/journal.pone.0223031)
Supplement: S2 Table — AA: arachidonic acid– 20:4n-6, EPA: eicosapentaenoic acid– 20:5n-3, DHA: docosahexaenoic acid– 22:6n-3; DMA: dimethylacetals. Data are reported as average of three replicates ± SD. Statistical differences are reported in comparison to ShellPaste. FA evidenced by SIMPER and differing significantly between diets are in bold. Letters correspond to statistical significance: a p>0.05, b p<0.05, c p<0.01, d p<0.001 (†). (DOCX) [file pone.0223031.s011.docx]

**S2_Table: Fatty acids composition of the five diets employed in this study, reported as percentage of each FAME for the total fatty acid content for each diet (% total FA) and as absolute fame content µg of FA per mg of algae dry weight (µgFA mg_DW_^-1^).** AA: arachidonic acid – 20:4n-6, EPA: eicosapentaenoic acid – 20:5n-3, DHA: docosahexaenoic acid – 22:6n-3; DMA: dimethylacetals. Data are reported as average of three replicates ± SD. Statistical differences are reported in comparison to ShellPaste. FA evidenced by SIMPER and differing significantly between diets are in **bold**. Letters correspond to statistical significance: a p>0.05, b p<0.05, c p<0.01, d p<0.001 (**†**).

| **FA Class** |  | ***C. fusiformis CCAP 1017/2*** | | | |  |  | ***I. galbana CCAP 927/1*** | | | |  |  | ***M. subterranean CCAP 848/1*** | | | |  |  | ***N. oceanica CCAP 849/10*** | | | |  |  | **ShellPaste** | | | |  |
| --- | --- | --- | --- | --- | --- | --- | --- | --- | --- | --- | --- | --- | --- | --- | --- | --- | --- | --- | --- | --- | --- | --- | --- | --- | --- | --- | --- | --- | --- | --- |
|  | **% total FA** | | | **µgFA mg_DW_^-1^** | | | **% total FA** | | | **µgFA mg_DW_^-1^** | | | **% total FA** | | | **µgFA mg_DW_^-1^** | | | **% total FA** | | | **µgFA mg_DW_^-1^** | | | **% total FA** | | | **µgFA mg_DW_^-1^** | | |
| **14:0** | **6.75^a^** | | **±0.54** | **6.73^a^** | **±0.66** | | **18.57^d^** | | **±0.30** | **16.47^d^** | **±2.12** | | **0.26^d^** | | **±0.01** | **0.26^d^** | **±0.02** | | **4.12^c^** | | **±1.22** | **4.04^a^** | **±1.85** | | **6.62^a^** | | **±0.79** | **4.89^a^** | **±1.48** | |
| 15:0 | 0.47^a^ | | ±0.02 | 0.47^a^ | ±0.08 | | 0.26^b^ | | ±0.02 | 0.23^a^ | ±0.01 | | 0.49^a^ | | ±0.22 | 0.48^a^ | ±0.20 | | 0.31^b^ | | ±0.05 | 0.30^a^ | ±0.09 | | 0.62^a^ | | ±0.09 | 0.46^a^ | ±0.13 | |
| 16:0 | 18.50^a^ | | ±2.07 | 18.43^a^ | ±2.54 | | 10.15^a^ | | ±0.11 | 8.99^a^ | ±0.98 | | 19.31^a^ | | ±0.99 | 19.06^a^ | ±1.33 | | 18.68^a^ | | ±6.74 | 18.33^a^ | ±9.51 | | 13.77^a^ | | ±1.38 | 10.16^a^ | ±2.73 | |
| 18:0 | 0.33^b^ | | ±0.05 | 0.33^a^ | ±0.08 | | 0.49^a^ | | ±0.16 | 0.44^a^ | ±0.20 | | 0.23^c^ | | ±0.04 | 0.23^a^ | ±0.02 | | 0.59^a^ | | ±0.28 | 0.58^a^ | ±0.34 | | 0.73^a^ | | ±0.11 | 0.54^a^ | ±0.17 | |
| **16:1n-9** | **4.04^b^** | | **±0.89** | **4.11^a^** | **±1.36** | | **2.93^c^** | | **±0.10** | **2.60^b^** | **±0.36** | | **10.67^a^** | | **±0.45** | **10.53^c^** | **±0.50** | | **4.97^a^** | | **±1.72** | **4.61a** | **±1.47** | | **7.31^a^** | | **±0.89** | **5.30^a^** | **±0.89** | |
| **16:1n-7** | **19.61^d^** | | **±1.58** | **19.55^d^** | **±2.37** | | **2.14^d^** | | **±0.24** | **1.89^c^** | **±0.21** | | **2.31^d^** | | **±0.28** | **2.27^c^** | **±0.12** | | **22.03^d^** | | **±1.07** | **20.86^d^** | **±3.55** | | **10.95^a^** | | **±1.23** | **8.05^a^** | **±2.02** | |
| **18:1n-9** | **3.24^a^** | | **±1.82** | **3.07^a^** | **±1.18** | | **11.34^b^** | | **±0.23** | **10.06^c^** | **±1.34** | | **1.30^b^** | | **±0.18** | **1.27^b^** | **±0.08** | | **4.51^a^** | | **±2.72** | **4.40^a^** | **±3.23** | | **6.03^a^** | | **±1.37** | **4.41^a^** | **±1.35** | |
| 18:1n-7 | 0.94^a^ | | ±0.45 | 0.96^a^ | ±0.53 | | 2.37^b^ | | ±0.56 | 2.09^b^ | ±0.46 | | 4.47^d^ | | ±0.72 | 4.46^d^ | ±1.10 | | 0.50^a^ | | ±0.09 | 0.47^a^ | ±0.06 | | 1.23^a^ | | ±0.03 | 0.89^a^ | ±0.15 | |
| 20:1n-11 | 0.41^a^ | | ±0.67 | 0.46^a^ | ±0.74 | | 9.22^d^ | | ±1.02 | 8.22^a^ | ±1.78 | | 0.02^b^ | | ±0.03 | 0.02^a^ | ±0.04 | | 0.02^b^ | | ±0.04 | 0.02^a^ | ±0.03 | | 0.86^a^ | | ±0.40 | 0.61^a^ | ±0.23 | |
| 20:1n-9 | 0.88^a^ | | ±1.30 | 0.97^a^ | ±1.44 | | 0.00^a^ | | ±0.00 | 0.00^a^ | ±0.00 | | 0.13^a^ | | ±0.17 | 0.14^a^ | ±0.19 | | 0.16^a^ | | ±0.21 | 0.13^a^ | ±0.15 | | 0.30^a^ | | ±0.04 | 0.22^a^ | ±0.01 | |
| 20:1n-7 | 0.03^a^ | | ±0.03 | 0.04^a^ | ±0.03 | | 0.00^a^ | | ±0.00 | 0.00^a^ | ±0.00 | | 0.00^a^ | | ±0.00 | 0.00^a^ | ±0.00 | | 0.06^a^ | | ±0.05 | 0.05^c^ | ±0.05 | | 0.07^a^ | | ±0.01 | 0.05^a^ | ±0.01 | |
| 22:1n-11 | 0.08^a^ | | ±0.14 | 0.08^a^ | ±0.15 | | 0.00^a^ | | ±0.00 | 0.00^a^ | ±0.00 | | 0.15^a^ | | ±0.17 | 0.15^a^ | ±0.18 | | 0.19^a^ | | ±0.24 | 0.16^a^ | ±0.18 | | 0.21^a^ | | ±0.14 | 0.15^a^ | ±0.09 | |
| 22:1n-9 | 0.00^d^ | | ±0.00 | 0.00^c^ | ±0.00 | | 0.08^a^ | | ±0.02 | 0.08^a^ | ±0.03 | | 0.00^d^ | | ±0.00 | 0.00^c^ | ±0.00 | | 0.02^c^ | | ±0.03 | 0.01^c^ | ±0.02 | | 0.16^a^ | | ±0.05 | 0.11^a^ | ±0.03 | |
| 18:2n-6 | 1.58^b^ | | ±0.61 | 1.53^a^ | ±0.32 | | 5.27^b^ | | ±0.21 | 4.66^c^ | ±0.42 | | 4.63^a^ | | ±0.51 | 4.54^c^ | ±0.14 | | 2.31^a^ | | ±0.24 | 2.17^a^ | ±0.19 | | 3.43^a^ | | ±0.83 | 2.49^a^ | ±0.63 | |
| **18:3n-6** | **2.64^d^** | | **±0.36** | **2.61^c^** | **±0.07** | | **0.13^a^** | | **±0.06** | **0.11^a^** | **±0.04** | | **4.69^d^** | | **±0.15** | **4.64^d^** | **±0.47** | | **0.09^a^** | | **±0.03** | **0.08^a^** | **±0.02** | | **0.07^a^** | | **±0.04** | **0.05^a^** | ±**0.03** | |
| 20:2n-6 | 0.00^d^ | | ±0.00 | 0.00^b^ | ±0.00 | | 0.14^a^ | | ±0.01 | 0.12^a^ | ±0.02 | | 0.00^d^ | | ±0.00 | 0.00^b^ | ±0.00 | | 0.10^a^ | | ±0.04 | 0.10^a^ | ±0.05 | | 0.11^a^ | | ±0.02 | 0.08^a^ | ±0.01 | |
| 20:3n-6 | 1.08^d^ | | ±0.40 | 1.04^c^ | ±0.20 | | 0.10^a^ | | ±0.01 | 0.09^a^ | ±0.02 | | 0.00^a^ | | ±0.00 | 0.00^a^ | ±0.00 | | 0.45^c^ | | ±0.07 | 0.43^b^ | ±0.11 | | 0.04^a^ | | ±0.04 | 0.03^a^ | ±0.03 | |
| **AA** | **9.05^d^** | | **±0.44** | **9.11^d^** | **±1.66** | | **0.10^c^** | | **±0.02** | **0.09^a^** | **±0.02** | | **0.00^d^** | | **±0.00** | **0.00^a^** | **±0.00** | | **5.20^d^** | | **±0.36** | **4.90^d^** | **±0.57** | | **0.46^a^** | | **±0.03** | **0.34^a^** | ±**0.07** | |
| 22:4n-6 | 0.00^a^ | | ±0.00 | 0.00^a^ | ±0.00 | | 0.25^d^ | | ±0.01 | 0.22^b^ | ±0.01 | | 0.00^a^ | | ±0.00 | 0.00^a^ | ±0.00 | | 0.00^a^ | | ±0.00 | 0.00^a^ | ±0.00 | | 0.02^a^ | | ±0.04 | 0.01^a^ | ±0.02 | |
| 22:5n-6 | 0.42^a^ | | ±0.08 | 0.42^a^ | ±0.06 | | 1.75^a^ | | ±1.59 | 1.51^a^ | ±1.32 | | 0.00^a^ | | ±0.00 | 0.00^c^ | ±0.00 | | 0.04^a^ | | ±0.04 | 0.04^b^ | ±0.04 | | 2.77^a^ | | ±0.59 | 2.02^a^ | ±0.57 | |
| **18:3n-3** | **0.15^d^** | | **±0.10** | **0.16^d^** | **±0.12** | | **7.19^b^** | | **±0.37** | **6.37^b^** | **±0.74** | | **26.63^d^** | | **±1.02** | **26.28^d^** | **±1.42** | | **0.11^d^** | | **±0.04** | **0.10^d^** | **±0.03** | | **4.48^a^** | | **±1.44** | **3.23^a^** | ±**0.99** | |
| **18:4n-3** | **0.98^b^** | | **±0.33** | **0.95^b^** | **±0.16** | | **9.86^a^** | | **±0.49** | **8.71^a^** | **±0.66** | | **2.99^a^** | | **±2.54** | **3.03^a^** | **±2.67** | | **0.05^d^** | | **±0.04** | **0.04^c^** | **±0.04** | | **7.50^a^** | | **±1.86** | **5.39^a^** | **±1.12** | |
| 20:4n-3 | 0.61^a^ | | ±0.38 | 0.57^a^ | ±0.26 | | 0.00^c^ | | ±0.00 | 0.00^c^ | ±0.00 | | 0.00^c^ | | ±0.00 | 0.00^c^ | ±0.00 | | 0.10^a^ | | ±0.03 | 0.09^a^ | ±0.02 | | 0.27^a^ | | ±0.03 | 0.20^a^ | ±0.05 | |
| **EPA** | **17.05^a^** | | **±2.74** | **17.34^a^** | **±4.91** | | **0.78^d^** | | **±0.17** | **0.70^d^** | **±0.23** | | **0.04^d^** | | **±0.07** | **0.04^d^** | **±0.07** | | **32.79^c^** | | **±7.57** | **30.67^d^** | **±6.77** | | **16.17^a^** | | **±0.75** | **11.86^a^** | ±**2.73** | |
| 22:5n-3 | 0.14^a^ | | ±0.03 | 0.14^a^ | ±0.02 | | 1.01^a^ | | ±1.60 | 0.90^d^ | ±1.43 | | 0.00^a^ | | ±0.00 | 0.00^a^ | ±0.00 | | 0.00^a^ | | ±0.00 | 0.00^a^ | ±0.00 | | 0.10^a^ | | ±0.02 | 0.07a | ±0.02 | |
| **DHA** | **0.79^d^** | | **±0.09** | **0.80^d^** | **±0.19** | | **12.69^d^** | | **±0.28** | **11.27^d^** | **±1.57** | | **0.05^d^** | | **±0.08** | **0.05^d^** | **±0.09** | | **0.00^d^** | | **±0.00** | **0.00^d^** | **±0.00** | | **6.17^a^** | | **±1.63** | **4.47^a^** | ±**1.25** | |
| 16;2 | 1.31^c^ | | ±0.28 | 1.33^a^ | ±0.44 | | 1.75^a^ | | ±0.22 | 1.57^a^ | ±0.37 | | 0.73^d^ | | ±0.06 | 0.72^a^ | ±0.07 | | 0.35^d^ | | ±0.20 | 0.32^b^ | ±0.19 | | 2.51^a^ | | ±0.24 | 1.85^a^ | ±0.45 | |
| **16;3** | **6.65^d^** | | **±1.40** | **6.79^d^** | **±2.23** | | **0.45^d^** | | **±0.33** | **0.38^b^** | **±0.25** | | **1.74^a^** | | **±0.16** | **1.72^a^** | **±0.19** | | **0.40^d^** | | **±0.15** | **0.37^b^** | **±0.07** | | **2.57^a^** | | **±0.53** | **1.86^a^** | **±0.41** | |
| 16;4 | 0.70^a^ | | ±0.62 | 0.76^a^ | ±0.67 | | 0.15^a^ | | ±0.01 | 0.13^a^ | ±0.02 | | 10.53^a^ | | ±9.14 | 10.66^a^ | ±9.42 | | 0.08^a^ | | ±0.07 | 0.07^a^ | ±0.07 | | 1.91^a^ | | ±0.38 | 1.40^a^ | ±0.36 | |
| 16:0 DMA | 1.18^b^ | | ±0.24 | 1.20^a^ | ±0.37 | | 0.82^c^ | | ±0.02 | 0.72^b^ | ±0.09 | | 3.09^a^ | | ±0.01 | 3.05^c^ | ±0.26 | | 1.49^a^ | | ±055 | 0.00^a^ | ±0.00 | | 2.26a | | ±0.33 | 1.65^a^ | ±0.35 | |
| *Σ*  SFA | 26.11^a^ | | ±2.10 | 26.02^b^ | ±2.93 | | 29.49^a^ | | ±0.37 | 26.16^b^ | ±3.32 | | 20.29^a^ | | ±0.88 | 20.02^a^ | ±1.20 | | 23.81^a^ | | ±8.18 | 23.34^a^ | ±11.7 | | 22.01^a^ | | ±1.82 | 16.24^a^ | ±4.43 | |
| ***Σ* MUFA** | **29.24^a^** | | **±1.74** | **29.25^a^** | **±4.18** | | **28.08^a^** | | **±0.57** | **24.94^a^** | **±3.44** | | **19.06^d^** | | **±0.39** | **18.86^a^** | **±1.85** | | **32.47^c^** | | **±2.37** | **30.70^a^** | **±5.15** | | **27.12^a^** | | **±0.30** | **19.80^a^** | **±3.69** | |
| ***Σ* n-6** | **14.77^d^** | | **±1.17** | **14.70^d^** | **±1.24** | | **7.74^a^** | | **±1.53** | **6.80^a^** | **±1.15** | | **9.31^b^** | | **±0.49** | **9.18^c^** | **±0.35** | | **8.19^a^** | | **±0.56** | **7.71^a^** | **±0.88** | | **6.91^a^** | | **±0.33** | **5.02^a^** | **±0.77** | |
| *Σ*  n-3 | 19.72^c^ | | ±2.29 | 19.95^a^ | ±4.81 | | 31.53^a^ | | ±1.96 | 27.95^a^ | ±3.66 | | 29.70^a^ | | ±1.79 | 29.41^a^ | ±3.61 | | 33.21^a^ | | ±7.65 | 31.04^a^ | ±6.77 | | 34.70^a^ | | **±**2.71 | 25.23^a^ | ±4.36 | |
| *Σ* PUFA | 43.15^a^ | | ±3.91 | 43.53^a^ | ±9.39 | | 41.61^a^ | | ±0.84 | 36.84^a^ | ±3.66 | | 52.02^a^ | | ±10.7 | 51.70^a^ | ±13.0 | | 42.23^a^ | | ±8.42 | 39.51^a^ | ±7.32 | | 48.61^a^ | | **±**1.90 | 35.36^a^ | ±5.75 | |
| **† equal letters indicate similar significance level: a: p>0.05; b: p<0.05; c: p<0.01; d: p<0.001.** | | | | | | | | | | | | | | | | | | | | | | | | | | | | | | |
