# Supplementary material for: Lipidomics analysis of juveniles’ blue mussels (Mytilus edulis L. 1758), a key economic and ecological species
Source: PLoS One. 2020 Feb 21;15(2):e0223031. doi: 10.1371/journal.pone.0223031 (PMC7034892; doi:10.1371/journal.pone.0223031)
Supplement: Attachment — Submitted filename: PONE-D-19-25119.doc [file pone.0223031.s015.doc]

**Manuscript number:** PONE-D-19-25119

**Title:** Lipidomics analysis of juveniles’ blue mussels (Mytilus edulis L. 1758), a key economic and ecological species

The authors represented a very good manuscript. Although the experimental results presented in this manuscript appears to be publishable, the manuscript needs to be minor revision. Therefore, it is not recommended for publication at its current state. My comments in details are as the following:

- Generally, Latin binomial must be used by authority in whole manuscript. Author name should not write italic according to Nomenclature. All author names should be added. In first stated section, species name should be written as *Mytilus edulis* L. and in other sections it should be written as *M. edulis* L. name should be written in stead of Mytilus sp.
- In abstract:

Methods should be written briefly. Also result section in the abstract should be written in an informative style. Please give real values/data, not vague subjective terms and avoid generalizations and nonessential information in the results.

Abbreviations such as GR, WI, PUFA etc. should be written clearly.

- In Material and methods section:

Abbreviations such as SAMS, FAME etc. should be written clearly.

- In result section:

Please correct “Table 3Error! Reference source not found.” in line 223 and “Error! Reference source not found.” in line 369.

- Conclusion section is very long. Authors should rewrite that part briefly.
