# Supplementary material for: Lipidomics analysis of juveniles’ blue mussels (Mytilus edulis L. 1758), a key economic and ecological species
Source: PLoS One. 2020 Feb 21;15(2):e0223031. doi: 10.1371/journal.pone.0223031 (PMC7034892; doi:10.1371/journal.pone.0223031)
Supplement: Attachment — Submitted filename: Response_to_reviewers_2020.01.docx [file pone.0223031.s016.docx]

6. Review Comments to the Author

Please use the space provided to explain your answers to the questions above. You may also include additional comments for the author, including concerns about dual publication, research ethics, or publication ethics. (Please upload your review as an attachment if it exceeds 20,000 characters)

Reviewer #1: The author has revised the manuscript according to all recommended corrections. The manuscript is acceptable.

Reviewer #2: The authors provided well responses for my comments and suggestions. Now I think the revised manuscript is acceptable for publication. The conlusion is still too long, please compress the content again.

We thank the reviewer for this comment; the conclusions were compressed and text reduced as requested.

Some errors need to corrected, for example:
Line 26-28, I think the subject was missed in the sentence.

We thank the reviewer for this comment; the text was amended accordingly.

From line 33，the are several subscript "ashfreeDW". What is it mean?

We thank the reviewer for this comment; ashfreeDW is the measure unit used throughout the study for absolute quantification of lipids. ashfreeDW refers to ash free dry weight (DW). The method to calculate the ash free DW is reported in section 2.2 and the acronym DW is now fully explained in the abstract.

line 54: "nutrients’ bioassimilation" should be "bioassimilation of nutrients"

We thank the reviewer for this comment; the text has been changed accordingly.

Line 143: "ml" should be "mL”. All "ml" should be "mL” , "ul" should be "uL" in the whole manuscript.

We thank the reviewer for spotting this out; all unit of measure have been corrected.

Line 186: what is the brand and model of the GC?

We thank the reviewer for this comment; text was modified accordingly

line 206: There are some errors in this line.

We thank the reviewer for this comment; the text has been corrected.

line 238, 310, 312: table 3 should be in one page.

We thank the reviewer for this comment, on our word processor table 3 is contained all at the bottom of one page. The journal submission guidelines requested to locate the table directly in the main manuscript text, in the paragraph right after the first in-text citation.
